# The Spatiotemporal Program of DNA Replication Is Associated with Specific Combinations of Chromatin Marks in Human Cells

**DOI:** 10.1371/journal.pgen.1004282

**Published:** 2014-05-01

**Authors:** Franck Picard, Jean-Charles Cadoret, Benjamin Audit, Alain Arneodo, Adriana Alberti, Christophe Battail, Laurent Duret, Marie-Noelle Prioleau

**Affiliations:** 1 Laboratoire Biométrie et Biologie Evolutive, UMR CNRS 5558 Univ. Lyon 1, Villeurbanne, France; 2 Institut Jacques Monod, CNRS, University Paris 7, Équipe Labellisé Ligue Contre le Cancer, Paris, France; 3 Université de Lyon, Laboratoire de Physique, UMR CNRS 5672 ENS Lyon, Lyon, France; 4 Génoscope-CEA Sequencing Center, Evry, France; Netherlands Cancer Institute, Netherlands

## Abstract

The duplication of mammalian genomes is under the control of a spatiotemporal program that orchestrates the positioning and the timing of firing of replication origins. The molecular mechanisms coordinating the activation of about 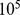 predicted origins remain poorly understood, partly due to the intrinsic rarity of replication bubbles, making it difficult to purify short nascent strands (SNS). The precise identification of origins based on the high-throughput sequencing of SNS constitutes a new methodological challenge. We propose a new statistical method with a controlled resolution, adapted to the detection of replication origins from SNS data. We detected an average of 80,000 replication origins in different cell lines. To evaluate the consistency between different protocols, we compared SNS detections with bubble trapping detections. This comparison demonstrated a good agreement between genome-wide methods, with 65% of SNS-detected origins validated by bubble trapping, and 44% of bubble trapping origins validated by SNS origins, when compared at the same resolution. We investigated the interplay between the spatial and the temporal programs of replication at fine scales. We show that most of the origins detected in regions replicated in early S phase are shared by all the cell lines investigated whereas cell-type-specific origins tend to be replicated in late S phase. We shed a new light on the key role of CpG islands, by showing that 80% of the origins associated with CGIs are constitutive. Our results further show that at least 76% of CGIs are origins of replication. The analysis of associations with chromatin marks at different timing of cell division revealed new potential epigenetic regulators driving the spatiotemporal activity of replication origins. We highlight the potential role of H4K20me1 and H3K27me3, the coupling of which is correlated with increased efficiency of replication origins, clearly identifying those marks as potential key regulators of replication origins.

## Author Summary

Replication is the mechanism by which genomes are duplicated into two exact copies. Genomic stability is under the control of a spatiotemporal program that orchestrates both the positioning and the timing of firing of about 50,000 replication starting points, also called replication origins. Replication bubbles found at origins have been very difficult to map due to their short lifespan. Moreover, with the flood of data characterizing new sequencing technologies, the precise statistical analysis of replication data has become an additional challenge. We propose a new method to map replication origins on the human genome, and we assess the reliability of our finding using experimental validation and comparison with origins maps obtained by bubble trapping. This fine mapping then allowed us to identify potential regulators of the replication dynamics. Our study highlights the key role of CpG Islands and identifies new potential epigenetic regulators (methylation of lysine 4 on histone H4, and tri-methylation of lysine 27 on histone H3) whose coupling is correlated with an increase in the efficiency of replication origins, suggesting those marks as potential key regulators of replication. Overall, our study defines new potentially important pathways that might regulate the sequential firing of origins during genome duplication.

## Introduction

The faithful duplication of mammalian genomes at each S phase is under the control of a spatiotemporal program that orchestrates and regulates both the positioning and the timing of firing of replication starting points also called replication origins. The molecular mechanisms involved in coordinating of the activation of 50,000 to 100,000 origins in each cell and at each cell cycle are still poorly understood, despite the need for a comprehensive understanding of these processes. Indeed, defects in the normal sequence of events leading to replication initiation may be directly responsible for genomic instability and/or the deregulation of differentiation programs. Consequently, the first and necessary step towards understanding this regulation is to refine our vision of the spatiotemporal replication program. For this reason several laboratories have chosen to map both the spatial and temporal programs of replication, in different systems and cell lines.

The temporal program of replication has been successfully analyzed in many laboratories with no particular controversy. By contrast, attempts to identify replication origins remain a subject of passionate debate in the field, as the intrinsic rarity of replication bubbles makes it difficult to purify the genomic material. The most popular method for mapping replication starting points in mammals is the purification of short nascent strands (SNS). Several laboratories have demonstrated that this purification requires the use of the 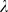-exonuclease to remove the high background due to broken genomic DNA [1]–[3]. The debate has been kept alive because previous studies that did not use 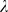-exonuclease [4] reported SNS levels incompatible with true initiation events [1], [5]. The debates then turned to the putative lack of overlap between datasets from different laboratories using SNS enrichment with 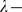
exonuclease purification, even for the same cell line, suggesting that the method was probably inaccurate. However this argument is almost entirely based on a comparison of two data-sets for the same cell line [1], [6], but the results of the first study [6] have repeatedly been shown to display a marked lack of overlap with those of other studies, whereas the results of the second [1] overlap significantly with those of other studies [7], [8].

Agreement on a consensual protocol for SNS enrichment and quantification has also become a critical issue as the scale of investigation of replication origins has changed profoundly in recent years. Beginning with investigations of individual loci, and continuing with the microarray technology, there has recently been another technological shift in this field towards the use of ultra-deep sequencing [8]. *Origin-omics* has now become a way of thinking about replication that incorporates tens of thousands of loci embedded within various genomic landscapes. The emphasis also needs to shift from protocols to methods used for the analysis of genome-wide replication data. Indeed, despite a spectacular increase in the sensitivity of detection, *Origin-omics* is already subject to the same pitfalls as all other types of *omics*: the difficulty achieving an appropriate balance between the specificity and sensitivity of the analysis method. In a recent study based on the ultra-deep sequencing of SNS, origins were detected using chIP-Seq tools [9] for peak detection. This resulted in 250,000 identified origins in different human cell lines [10]. These predictions cover 6% of the human genome, with average origins length of 760 bp, that presumably includes most of the previously reported origins (except those reported in the above mentioned study [6], which should now reasonably be excluded). We noticed however one possible caveat in the use of chIP-Seq tools for the detection of replication origins based on sequenced SNS. Indeed, *prior* to the sequencing, SNS are first selected based on their size (about 1.5–2 kb). Hence the resolution of detection of replication origins cannot be less than this size. It is therefore possible that chIP-Seq tools tend to split the signal into multiple peaks and hence tend to overestimate the number of replication origins. In this work, we first address this issue of resolution of detection. We propose a peak-detection method that is adapted to the special case of SNS sequencing data, based on the *prior* control of the resolution of detection of exceptional local enrichments of reads. The method relies on sliding windows, the size of which is imposed by the size of the sequenced SNS fragments. We deal with multiple testing by providing a significance threshold that controls for false-positive detections, and that is adaptive to local coverage variations. The consensus on the SNS purification protocol made it possible to apply our method to our samples (K562 cells) and to published data [10] (from four different cell lines), which allows us to compare detection methods on SNS data.

*Origin-omics* shares another difficulty with other *omics* fields, which is the need for validation of genome-wide detections by an independent method. Very interestingly, the field has recently been enriched by another genome-wide map of replication origins obtained by bubble trapping [11], which is based on the sequencing of EcoR1 fragment containing at least one replication bubble. This new map consists of ∼125,000 EcoR1 fragments that cover 25% of the human genome. We took this opportunity to confront different genome-wide detections of replication origins based on different methods and protocols, which had never been done before. Comparisons between SNS-based origins and bubble trapping based origins on different cell lines show a good agreement between maps. Furthermore these comparisons indicate that the sensitivity and specificity of the detection of origins based on SNS data is significantly improved with our dedicated method compared to previously used chIP-Seq tools.

Now that a consensus set of replication origins has been identified, the time has come to unravel the genomic and epigenetic characteristics that make these particular loci replication origins. To proceed we focus on the connections between the spatial and temporal programs of replication at fine scales. It is now well established that genomes are organized into early-, mid- and late-replicating domains, and early domains have been shown to be associated with active epigenetic marks such as H3K4me1, 2 and 3, H3K27ac, H3K36me3 and H3K9ac [12]. However, different studies have generated conflicting results, demonstrating the difficulties involved in precisely defining the chromatin landscape of the domains replicated in mid- and late S-phase. The first genome-wide studies showed that late-replicating domains were weakly correlated with the repressive mark H3K9me2, but not with H3K27me3 [12]. This result conflicted with the finding of a previous study based on 1% of the human genome (ENCODE regions), which reported a strong correlation between late replication and H3K27me3 [13]. Finally, an association of H3K27me3 with mid-S phase-replicating chromosomal domains was recently demonstrated, together with a substantial correlation with early-replicating domains [14]. These results also highlight the difficulties involved in assessing the impact of specific modifications on normal S-phase progression. We hypothesize that the imprecise mapping of origin positions has hampered the search for specific epigenetic signatures. In this study we integrated data collected in several genome-wide studies aiming to map DNA replication timing domains and chromatin states. We provide unique datasets including origin position, efficiency and timing, and the local genomic characteristics of each origin (histone modifications, sequence characteristics). Overall, our findings make it possible to define new mechanisms potentially involved in defining of origin sub types activated sequentially during S phase.

## Results

### Sliding windows for the detection of significant read enrichments

**Figure 1:**
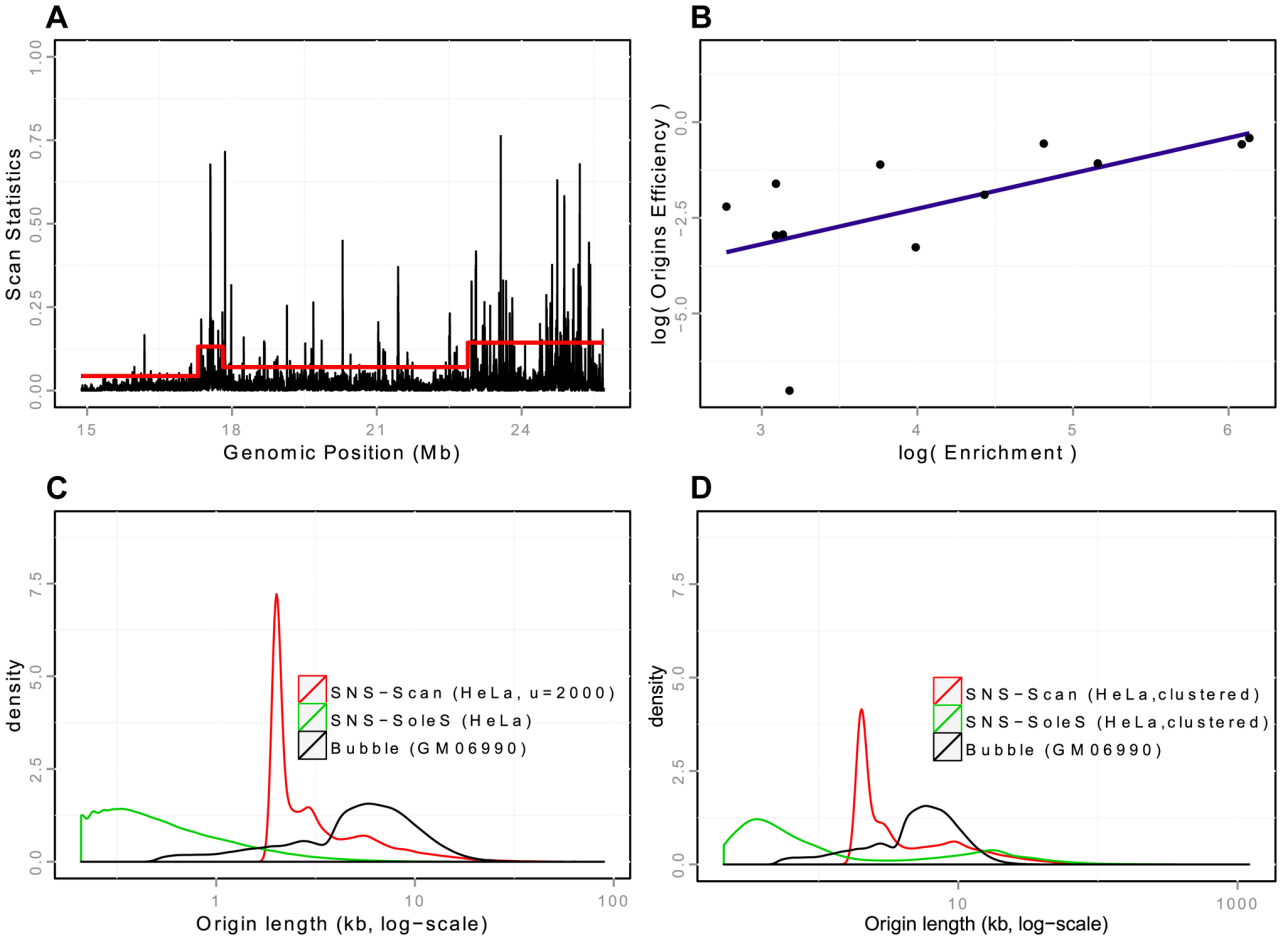
Detection and validation of Ori-Seq peaks. A: Snapshot of peak detection on a region of chromosome 20 from HeLa cells. Scanning windows are represented according to their genomic position. The red line corresponds to the threshold used in the scan methods, which adapts to regional coverage. B: Quantification property of the OriSeq method (scan-based detections): Origin efficiency is defined as the number of reads within an origin divided by the length of the origin. A correlation of 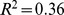 is observed between origin efficiency as defined by OriSeq and origin efficiency, as assessed by qPCR (on a log scale). C: Distributions of origins length (log-scales) for different protocols (SNS, Bubble trapping), different methods of detection (scan, SoleSearch),on HeLa cells for SNS-ori and GM06990 for Bubble ori. D: Distributions of origins length (log-scales) for clustered SNS origins, compared with the distribution of the length of Bubble origins.

OriSeq data analysis based on SNS material consists in detecting significant read enrichments corresponding to accumulations of SNS throughout the human genome. For a given origin, reads accumulate around the initiation starting point with a span determined by the size of the SNS fragments. It is important to notice that SNS are selected based on their size (between 1.5–2 kb), and then fragmented and sequenced. hence, for a given origin the resolution of detection can not be smaller than 1.5–2 kb. Tools for the detection of peak-like patterns in ChIP-Seq data, such as SoleSearch [9], [10], have been used for detection purposes, without controlling for the size of the peak, which results in peaks smaller than 1 kb on average (Table 1). In our method we control the resolution of detection by considering sliding windows of size 2 kb. Then we define an appropriate statistical model for discriminating between signal and noise and controlling for false-positive peaks, while accounting for the genome-ordered structure of the data. We used scan statistics by calculating the probability that the richest window corresponded to a false positive [15]. This approach is designed to avoid false-positive detections, and was calibrated adaptively to coverage variations to account for coverage heterogeneities along the genome (Figure 1-A). Using the scan method, we detected between 60,000 and 90,000 replication origins (depending on read depth), which cover ∼12% of the genome (Table 1). Details are provided in the Methods Section.

**Table 1:** Global view of origins datasets and detections characteristics.

cell-line	bp sequenced (Mb)	nb. oris	ave. length (kb)	cum. length (Mb)	% genome covered
SNS-SoleS origins [10]					
Hela	89	233,545	0.76	178	5.93
IMR90	85	256,990	0.74	190	6.33
iPS	87	246,866	0.72	179	5.95
H9	84	208,520	0.82	171	5.69
SNS-scan origins					
Hela	89	90,073	3.92	353	11.77
IMR90	85	89,889	4.33	389	12.97
iPS	87	93,896	3.95	371	12.36
H9	84	79,556	3.88	309	10.29
K562	30	59,185	3.36	199	6.63
Bubble origins [11]					
GM06990	42	123,264	6.4	750	24.99

We first generated our SNS samples from K562 cell lines (see Materials and Methods) and we showed that the reproducibility of our detections was good, as 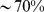 of origins detected in one technical replicate are found in another (on K562 cells, Supp. Table S1), which actually corresponds to the technical reproducibility of origins detected by bubble trapping [11]. Then we investigated the quantitative properties of our analysis. In OriSeq data, which are obtained from populations of asynchronous cells, the number of reads for a detected origin reflects the percentage of cell cycles using this locus as a starting point for replication. The density of reads within an origin, therefore, constitutes a measurement of the efficiency of that origin. We assessed the precision of our method, by randomly selecting weak, intermediate and strong origins on the basis of read densities. We found that the number of reads detected for a given origin and the efficiency of that origin, as assessed by qPCR on an independent SNS preparation, were correlated (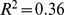, 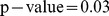, Figure 1-B). These experimental validations confirm that the set of replication origins detected by our method is likely to correspond to true positive initiation events.

### Comparing genome-wide detections of replication origins: A matter of resolution

To evaluate the reproducibility of replication origin detections, we compared our results with those of two previously published studies [10], [11]. The first dataset was obtained with the same SNS purification protocol but in a different laboratory [10], on different cell lines (IMR-90, HeLa, human embryonic stem cell H9, induced pluripotent stem cells from IMR90 (IPS)). These datasets were comparable with ours, despite coverage differences (Table 1). In the original publication, detections were made using SoleSearch on these data [9]. For comparison we re-analyzed them using the scan method (Table 1). The second dataset corresponds to origins detected using bubble trapping, which is based on the sequencing of EcoR1 fragments containing at least one bubble (on GM06990 cells). In this case detections were based on a background read depth distribution [11]. For the sake of simplicity, the three data sets will be referred to as SNS-scan, SNS-SoleS and Bubble origins.

The three methods differ widely in the number of detected origins, with about 2 to 3 times more SNS-SoleS origins than Bubble and SNS-scan origins, even for data from the same cell lines (Table 1). SNS-SoleS origins are 760 bp long on average and cover ∼6% of the genome, whereas SNS-scan origins are longer (∼4 kb on average) and cover ∼12% of the genome, and Bubble origins (6.4 kb long on average) cover ∼25% of the genome (Table 1, Figure 1-C). The strong contrast between SNS-detected origins and Bubble origins reflects differences in the level of resolution of the methods: whereas SNS data allow the detection of origins at relatively high resolution (based on 1.5–2 kb fragments), the resolution of bubble trapping experiments is limited by the genomic density in EcoR1 restriction sites. For SNS data, the main difference between our scan approach and the SoleSearch method is that this latter does not control the level of resolution and hence tends to detect many small peaks. To compare datasets we computed the proportion of origins of a given dataset that overlap with origins of another dataset.

### Comparison of peak detection methods on SNS data

In a first step we compared SNS-scan origins with SNS-SoleS origins on 4 different cell lines (Table 2). On average 70% of SNS-scan origins overlap with SNS-SoleS origins (on the same cell line) and 58% of SNS-SoleS origins overlap with SNS-scan origins, compared with 3–6% expected by chance (Table 2, the randomization procedure being detailed in the Methods section). Visual inspections suggested that in many cases, SNS-SoleS origins corresponded to multiple small peaks located within a same SNS-scan origin (Supp. Figure S1). To account for this difference in resolution, we clustered neighboring SNS-SoleS results so that origins from both methods have the same length on average (Table 2, Supp. Figure S1). By doing so, 71% SNS-scan origins overlap with (clustered) SNS-SoleS origins and 63% of SNS-SoleS origins overlap with SNS-scan origins. Thus, when compared at the same resolution, the overlap between methods is between 60 and 70%.

**Table 2:** Comparison of SNS-scan origins and SNS-SoleS origins.

Cell Line	# ori	ave. length (kb)	Cum length (Mb)	% genome covered	% Overlap			
					SoleS in scan		scan in SoleS	
					Obs.	Exp	Obs.	Exp
SNS-SoleS origins								
HeLa	233,545	0.76	178	6	71	6	59	3
IMR90	256,990	0.74	190	6	70	6	59	3
iPS	246,866	0.72	179	6	68	5	56	4
H9	208,520	0.82	171	6	73	5	57	3
Average	236,457	0.76	179	6	70	5	58	3
Clustered SNS-SoleS origins (12 kb apart)								
HeLa	156,952	3.2	509	17	71	5	63	4
IMR90	169,195	3.3	554	18	70	5	64	4
iPS	165,905	3.2	533	18	69	5	62	4
H9	144,904	3.1	445	15	73	4	62	4
Average	159,239	3.2	510	17	71	5	63	4

Overlap between SNS-Scan origins and SNS-SoleS origins [10]. % Overlap (SoleS in scan) corresponds to the number of scan origins that overlap with SoleS origins divided by the total number of scan origins. Expectations are assessed by randomly sampling genomic intervals on the mappable fraction of the human genome (see Materials and Methods). SNS-SoleS were clustered so that origins less than 12 kb apart were clustered into one single (larger) origin.

### Comparison of SNS and Bubble trapping detections

Then we compared SNS origins with Bubble origins to assess the overlap between experimental protocols. Here 45–46% of SNS origins (SNS-SoleS or SNS-scan) overlap with Bubble origins and 36–37% of Bubble origins overlap with SNS origins (*vs.* 5–7% expected by chance, Table 3). Given the strong difference in resolution between the two methods (Table 1, Supp. Figure S1), we repeated the comparison after having clustered SNS origins (so that to obtain a resolution comparable to that of Bubble origins (Table 3). With this procedure, 65% (51%) of SNS-scan (SNS-SoleS) origins overlap with Bubble origins and 44% (37%) of Bubble origins overlap with SNS-scan (SNS-SoleS) origins, compared to 6–7% expected by chance (Table 3). We note that the cross-validation of SNS-detected origins by Bubble origin data is stronger when we used SNS-scan origins than SNS-SoleS origins, which suggests that the scan approach achieves a better sensitivity and specificity than SoleSearch. It should be noticed that the comparisons between replication origins detected by SNS or by bubble trapping were performed on different cell lines, and hence underestimate the true overlap between the different methods. The matter of resolution appears central in a fair comparison between datasets, and was partly assessed by considering clustered SNS origins. However the distributions of origins length still differ between methods, despite comparable on average (Figure 1-D). Nevertheless, this study demonstrates a good agreement between SNS-based and bubble trapping-based replication origin maps, with at least 65% of SNS origins confirmed by bubble trapping.

**Table 3:** Comparison of SNS origins and Bubble origins.

Cell Line	# ori	ave. length (kb)	cum. Length (Mb)	% genome covered	% Overlap			
					B in SNS		SNS in B	
					Obs.	Exp	Obs.	Exp
SNS-SoleS origins								
Hela	233,545	0.76	178	6	46	6	37	7
IMR90	256,900	0.74	190	6	45	5	37	7
iPS	246,866	0.72	179	6	46	5	37	7
H9	208,520	0.82	171	6	46	5	33	7
Average	236,458	0.76	180	6	46	5	36	7
Clustered SNS-SoleS origins (20 kb apart)								
Hela	134,141	6.5	868	29	51	7	40	6
IMR90	144,811	6.5	938	31	50	6	40	6
iPS	141,514	6.5	917	30	51	6	40	6
H9	125,951	5.9	744	25	50	6	36	6
Average	136,554	6.3	867	26	51	6	39	6
SNS-scan origins ( 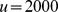 )								
Hela	90,073	3.9	353	12	46	5	37	6
IMR90	89,889	4.3	389	13	45	5	37	6
iPS	93,896	3.9	371	12	45	5	37	6
H9	79,556	3.9	309	10	45	5	32	6
Average	88,353	4	355	12	45	5	36	6
Clustered SNS-scan origins (6 kb apart)								
Hela	66,270	6.7	443	15	67	8	45	6
IMR90	66,679	7.1	476	16	65	7	45	6
iPS	69,396	6.7	463	15	65	7	46	6
H9	61,454	6.1	377	13	63	7	40	6
Average	65,950	6.7	440	15	65	7	44	6

Overlap between SNS origins Bubble origins [11]. % Overlap (B in SNS) corresponds to the number of SNS origins that overlap with Bubble origins divided by the total number of SNS origins. Expectations are assessed by randomly sampling genomic intervals on the mappable fraction of the human genome (see Materials and Methods). SNS-SoleS and SNS-scan were clustered so that origins that were 20 kb (resp 6 kb) apart were clustered into one single (larger) origin.

### Most early origins are constitutive, efficient, and strongly associated with CGIs

The first attempts to unravel the spatial program of replication showed that replication origins were associated with specific genomic features, such as high GC content, CpG islands (CGIs) [1], [16], [17] and G-quadruplexes [10], [18]. Moreover, a recent study showed that origins could be divided into subgroups [10]: constitutive origins, which are common to all cell lines investigated; specific origins, which are found in only one cell type; and common origins, which are neither constitutive nor specific. This suggests that the same genomic and epigenomic constraints may apply to all constitutive origins (and possibly to other types of origins too).

We confirmed the strong overlap of datasets from different cell lines, as constitutive origins accounted for 57%, 35%, and 35% of origins in K562, HeLa and IMR90 cell lines respectively, whereas specific origins accounted for only 15, 13, and 9%. We then showed that the clustering of origins previously described [10] was specific to constitutive origins, as the size distribution was skewed towards large sizes (average sizes of 4.1 kb, 2.4 kb, 2.1 kb for constitutive, common and K562-Specific Oris, respectively, Supp. Figure S2), indicating that constitutive origins may present the highest density of initiation events.

**Figure 2:**
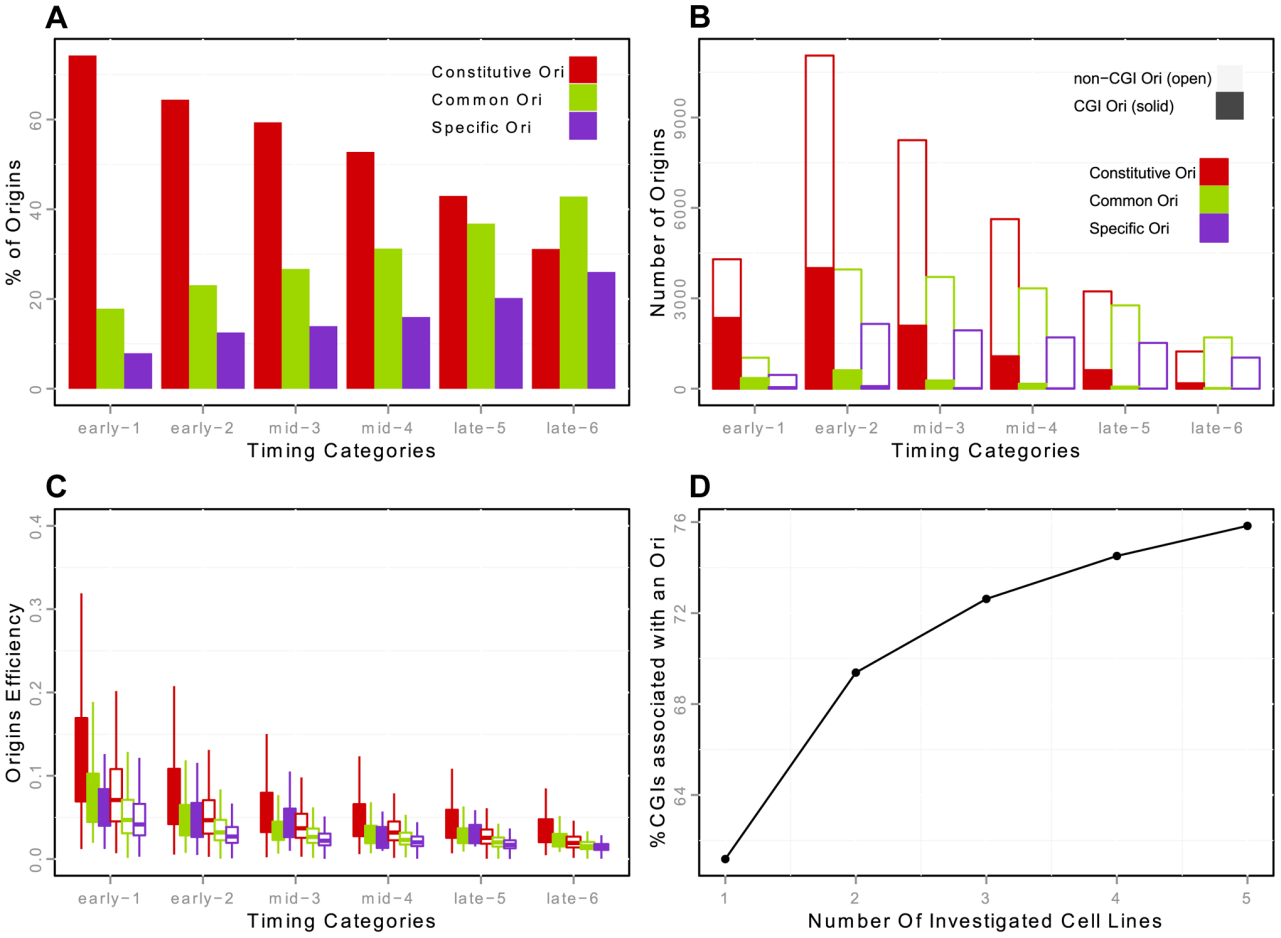
Constitutive, common and cell specific replication origins (K562) and association with CGIs. A: Percentage of constitutive/common/K562-specific origins in each timing category (from early to late origins, as explained in the Methods Section). Constitutive origins are determined by the intersection of the origins from the five cell lines -H9, HeLa, IMR90, K562 and iPS- with Galaxy [47]. B: Number of constitutive/common/K562-specific origins in each timing category, with distinction between origins associated and not associated with CGIs. Origins classified as CGIs correspond to origins strictly overlapping a CGI. Positions of CGIs are taken from UCSC Genome Browser annotation. C: Boxplots of origin efficiency according to timing, constitutive/common/K562-specific nature and association with CGIs. Origin efficiency is defined as the number of reads within the origin interval divided by the length of the origin (i.e. the density of reads within a given origin). D: Proportion of CGIs overlapping with an origin in at least one cell line, according to the number of cell lines analyzed.

We then connected the temporal and spatial programs of replication at fine scales, by assigning a temporal status to each origin on the basis of publicly available replication timing data [19]–[21], as explained in the Methods section. This made it possible to distinguish between origins activated in early S-phase (classes 1–2) and origins activated in mid- and late S-phase (classes 3–4 and 5–6, respectively). Most early origins (67%) were found to be constitutive, whereas late origins tended to be more cell type-specific (Figure 2-A for K562 cells, Supp. Figure S3-A for HeLa and IMR90 cells). This suggests that a large proportion of the origins replicated early in S phase are controlled by a highly robust combination of factors common to most cell lines.

We then focused on the strong overlap between replication origins and CGIs [1], [16], [17]. We showed that this enrichment was not homogeneous throughout S phase, as 32.5%, 15% and 8% of early, mid and late origins overlapped with CGIs, this enrichment being significant whatever the timing of replication, in the three cell lines investigated (Table 4). This suggests that CGIs intrinsically favor initiation activities. In addition, 86% of the origins associated with CGIs were constitutive origins (Figure 2-B, Supp. Figure S3-B). Read accumulation levels were much higher for CGI-constitutive origins (Figure 2-C, Supp. Figure S3-C), consistent with the larger size of the constitutive-CGI origins (Supp. Figure S2). Moreover, when considering origin efficiency, corresponding to the total number of reads divided by the length of the origin, origins were found to be more efficient in early S phase (as already reported [10], [16]), but we found that origins were more efficient when associated with CGIs (Figure 2-C, Supp. Figure S3-C).

**Table 4:** Association of replication origins with CGIs and TSSs, as a function of replication timing category.

		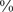 CGIs		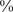 TSS	
K562	Number of Ori	Obs.	Exp.	Obs.	Exp.
1-early	5784 ( 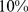 )	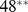	5	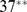	6
2-early	17164 ( 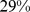 )	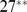	5	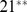	6
3-mid	13890 ( 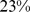 )	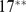	5	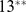	7
4-mid	10661 ( 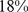 )	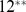	5	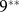	6
5-late	7513 ( 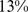 )	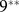	5	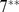	6
6-late	3971 ( 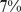 )	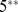	4	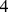	6

Number and percentage of origins in each timing category (from early to late). Origins classified as CGI (or TSS) correspond to origins that strictly overlap with a CGI (or TSS). The positions of CGIs and TSSs were taken from the UCSC Genome Browser annotation. Expected (Exp.) percentage and significance of association (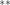 for significant enrichment, with 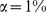) are computed with random genomic segments sampled from mappable regions (see Materials and Methods).

As most CGIs overlap with promoters and transcriptional regulatory elements, we also analyzed the distribution of origins with respect to transcription start sites (TSSs). We also used maps of several chromatin states as a function of transcriptional activity [22] to evaluate the impact of transcription on the replication program. We found that 5 to 37% of origins were associated with a TSS in K562 cells, depending on the timing of replication (Table 4). Inactive poised promoters were poorly represented, whereas active and weak promoters were evenly distributed and significantly enriched in early S origins (Table 5). The association of many origins with weakly transcribed regions (39% of early origins and 19% of mid-S phase origins were found to be associated with chromHMM-11, Table 5) is also consistent with previous studies [16], [23] indicating that many initiation events take place within the body of genes. We also found associations between early origins and strong and weak poised enhancers (Table 5).

**Table 5:** Association of replication origins with chromatin states and timing.

	chromHMM 1		chromHMM 2		chromHMM 3		chromHMM 4		ChromHMM 5	
	Active Prom.		Weak Prom.		Inactive/Poised Prom.		Strong Enh.		Strong Enh.	
K562	obs	Exp.	obs	Exp.	obs	Exp.	obs	Exp.	obs	Exp.
1-early	10 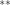	3	11 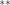	4	2 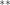	1	15 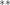	4	14 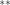	4
2-early	4 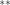	3	6 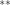	4	2 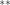	1	8 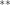	4	8 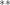	4
3-mid	2 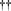	3	4	4	1	1	4	4	4	4
4-mid	1 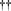	3	2 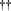	4	1	1	2 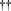	4	2 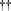	5
5-late	1 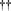	2	1 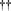	4	0 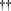	1	1 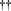	4	1 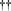	4
6-late	0 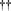	2	1 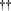	3	0	1	0 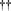	4	0 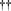	4

Origins associated with a given chromatin state (in percent) correspond to origins that overlap with published chromatin state coordinates [22], from the UCSC Genome Browser with chromHMM 1: active promoter, chromHMM 2: weak promoter, chromHMM 3: inactive/poised promoter, chromHMM 4: strong enhancer, chromHMM 5: strong enhancer, chromHMM 6: weak/poised enhancer, chromHMM 7: weak/poised enhancer, chromHMM 8: insulator, chromHMM 9: transcriptional transition, chromHMM 10: transcriptional elongation, chromHMM 11: weak transcribed, chromHMM 12: Polycomb-repressed, chromHMM 13: heterochromatin; low signal, chromHMM 14: repetitive/copy number variation, chromHMM 15: repetitive/copy number variation. The expected (Exp.) percentage and significance of association (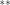 for significant enrichment, with 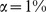 and 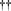 for significant depletion) were calculated with random genomic segments sampled from mappable regions (see Materials and Methods).

These results confirm the early data obtained with small fractions of the human and mouse genomes [1], [17] and indicate that the contribution of CGIs to the establishment of the spatiotemporal program of DNA replication extends to the whole genome, together with potential transcriptional regulatory elements. Moreover, the extension of the study to several cell lines showed that 42% of CGIs were very efficiently recognized as sites of replication initiation in all cell types, thus defining a very robust and efficient subset of replication origins with a tendency to fire early in S phase. The key role of CGIs was also highlighted by the clear increase in the percentage of CGIs overlapping at least one origin in one cell line with the number of cell lines investigated (Figure 2-D). With the datasets for five cell lines used here, we were able to associate ∼76% of CGIs with at least one origin, but the trend clearly suggests that most CGIs are potential origins of replication.

### Association with ORC1 binding

**Figure 3:**
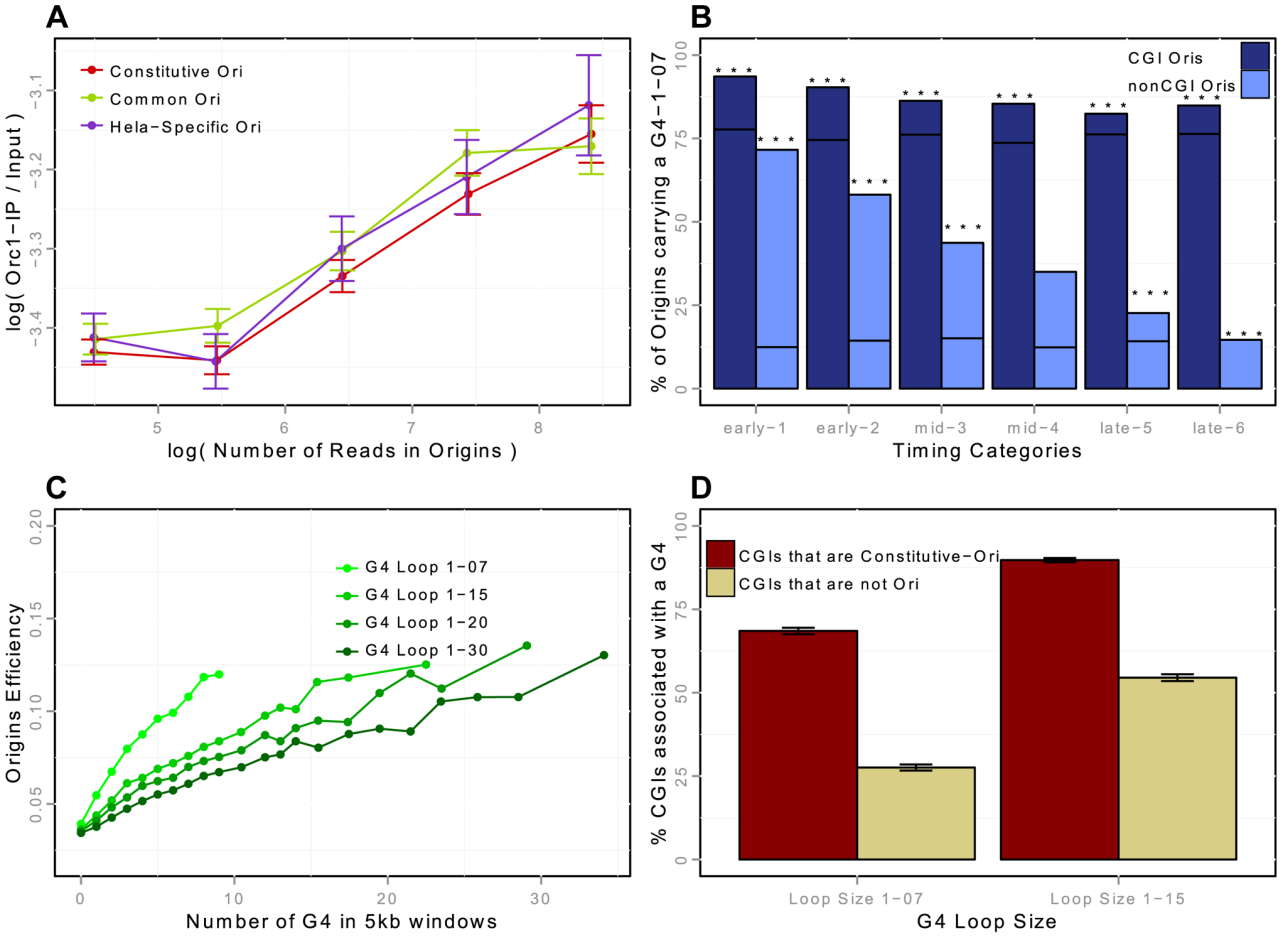
Association of replication origins with ORC1 (in HeLa cells) and with G-quadruplex motifs (in K562 cells). A: ORC1-chIP signal with respect to the number of reads in detected origins in HeLa cells (on a log scale). ORC1-chIP signal (y-axis) corresponds to the log-ratio of the number of reads in ORC1 peaks between the IP and the Input data. B: Percentage of replication origins (K562) associated with G-quadruplexes with a loop size of 1-07 according to the association with CGIs and timing categories (from early to late origins, as explained in the Methods Section). Notation G_4_1-07 in the figures corresponds to G-quadruplex motifs with loop sizes shorter than 7 nucleotides (G_4_N_1–7_G_4_N_1–7_G_4_N_1–7_G_4_). Black lines correspond to the expected percentage association of random segments with G-quadruplexes, and stars correspond to exceptional enrichment, with 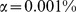. C: Impact of the density of G-quadruplexes of given loop sizes on the efficiency of replication origins. G-quadruplex motifs are detected with Quadparser [43], and origins are considered associated with a G-quadruplex if they overlap any G-quadruplex motif of a given loop size (from 7 to 30). Origin efficiency is defined as the of the number of reads within the origin interval divided by the length of the origin. D: Percentage of CGIs associated with a G-quadruplex of loop size 1-07 or 1-15, according to the association of CGIs with constitutive replication origins, or with no replication origin (CGIs-nonOri are CGIs that are not associated with replication origins in any of the 5 cell lines investigated).

ORC1 binding sites were recently mapped genome-wide in HeLa cells [7]. ORC1 is a subunit of the origin recognition complex (ORC) used as a landing platform for the assembly of a cascade of components that together form the prereplicative complex (pre-RC) in G1. Pre-RCs, including the core replicative helicase Mcm2–7, are sequentially activated during S phase. It has now been established that more pre-RCs are formed in G1 than are actually required in S phase. This redundancy has the advantage of providing “dormant origins”, which may be used in a fail-safe mechanism activated in the vicinity of arrested replication forks, to restart replication [24], [25]. Thus, this model predicts that more pre-RC (ORC1) binding sites than initiation sites should be mapped. However, only 13,604 ORC1-enriched peaks were identified [7], suggesting that the ORC1-ChIP experiment was not sensitive enough to detect every site of replication initiation and/or pre-RC formation. Nonetheless, the strong association with TSSs and known replication origins observed suggests that this new analysis identifies potential initiation sites (even though restricted to a subset). We, therefore, studied the association of ORC1 binding sites with replication origins observed in HeLa cells, and found that 44% (5,957) of ORC1 peaks were located within replication initiation sites (vs. 3%, as would be expected by chance). Given that the overlap between two anti-ORC1 ChIP-Seq replicates in HeLa cells was ∼60%, these results suggest that our SNS method is highly reliable for the determination of origin positions. The association of origins with ORC1 was stronger for constitutive origins (66% (3917) of ORC1 binding sites co-localize with constitutive origins, 30% and 4% for common and cell-specific origins respectively), consistent with the stronger Orc1-chIP signal for efficient origins (Figure 3-A). Consistently, ORC1-bound origins were found to be enriched in CGIs (60% are CGIs, and CGI-origins account for 16% of all origins), supporting a central role for CGIs in replication.

### Origins are strongly associated with G-quadruplexes

Two recent studies suggested that G-rich motifs capable of forming G-quadruplexes (G4s) are potential regulators of origin function [10], [18]. These motifs are able to form four-stranded DNA structures with loops of different sizes. We showed that the association of G4 with origins was dynamic and dependent on the association with CGIs: the enrichment in CGI-origins was higher than expected and remained high for origins of all replication timing groups, whereas the enrichment of non-CGI origins in G4 was also much higher than expected but was lower for late-replicating origins (Figure 3-B). We then assessed the impact of the size of the G4 loops on origin efficiency (Figure 3-C). We showed that the efficiency of replication origins increased with the local density of G4s (as measured by the number of G4s in a 5 kb window), and that G4s with short loops had a greater impact (Figure 3-C). This result is in agreement with the observation that on one model origin two G4 motifs cooperate to drive initiation very efficiently [26].

We investigated the importance of G4s for origin selection further, by determining whether CGIs associated with origins (Ori-CGIs) displayed a higher level of enrichment in G4s than CGIs not associated with origins (nonOri-CGIs). The human genome contains a total of 28,691 CGIs (from the UCSC database-hg19), 50% of which overlap with constitutive origins (76% overlapped with origins in at least one of the cell lines investigated). As mentioned above, constitutive origins are the most efficient and tend to fire in early S phase. We found that CGIs overlapping with constitutive origins displayed a greater enrichment in G4 L1–7 and L1–15 than nonOri-CGIs (Figure 3-D). This result again strongly supports the hypothesis that G4 L1–7 plays an important role in the control of origin selection.

### Replication origins interact dynamically with histone marks at fine scales

Many studies have tried to decipher the roles of histone marks and nucleosomal organization in origin selection. However, our understanding of the complex relationships between chromatin states and replication has been limited by the scale of investigation, as all studies consider replication timing domains of 200 kb to 2 Mb, potentially resulting in a lack of resolution [27]–[30]. Moreover, origins of replication have been found embedded within many types of chromatin substrates [1], [31], [32], suggesting that any regulatory effect of chromatin structure would not be homogeneous across replication initiation sites. This was confirmed by studies in mouse Embryonic stem cells (ESCs) and neural precursor cells (NPCs), showing regions that replicate early to be enriched in open chromatin marks, such as H3K4me3 and H3K36me3 [33], whereas little (if any) association was detected with other marks, such as H3K27me3, H3K9me3 or H4K20me3. Investigations of the methylation of H4K20 have provided new insight. Several studies have shown that PR-Set7, which is involved in depositing the histone mark H4K20me1, plays a role in the control of origin firing [34]. These findings are consistent with recent data indicating that Suv4-20h plays a crucial role in the further methylation of H4K20me1 [35]. Nevertheless the fraction of replication origins that really do carry this mark (and are therefore potentially regulated by this modification) remains unknown.

**Figure 4:**
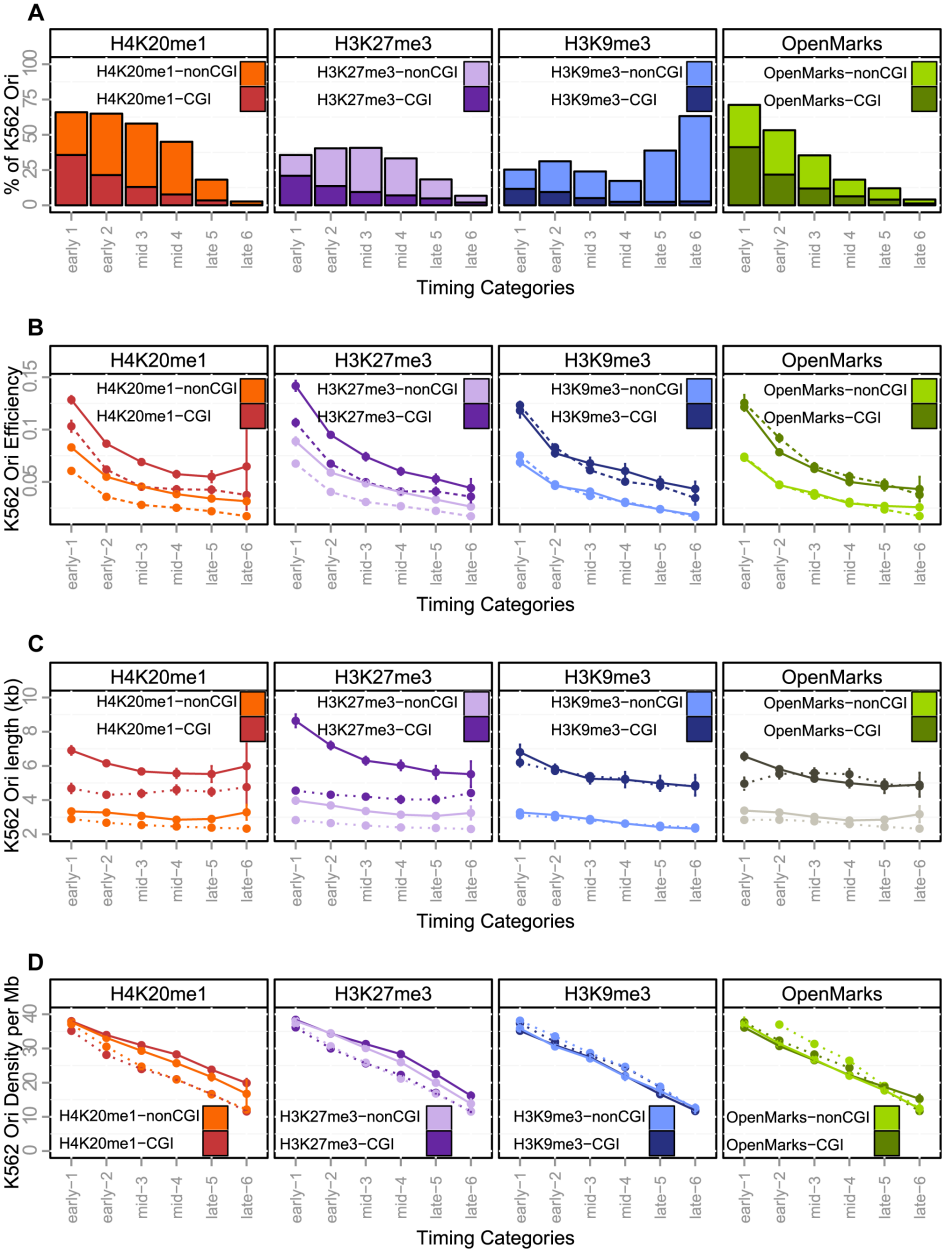
Effect of histone marks associations on origins efficiency, length and density (K562). A: Percentage of origins associated with chromatin marks according to timing categories (from early to late origins, as explained in the Methods Section) and CGI association. B: Variations of origin efficiency (as defined by the number of reads divided by the length of the origin) with replication timing, mark associations, and association with CGIs. C: Variations of origin length in kb with replication timing, mark associations, and association with CGIs. D: Variations of origins density (as defined by the number of origins per Megabase) with replication timing, mark associations, and association with CGIs. Values correspond to mean values with 95% confidence intervals. A comparison of the solid and dashed lines highlights the effect of associations of marks. OpenMarks indicates origins associated with either H2AZ, H3K9ac or H3K4me3.

Our study provides a unique framework for unraveling the connections between the fine-scale spatiotemporal program of replication and the landscape of chromatin modifications (links to chromatin data are provided in Supp. Table S3). The H4K20 monomethylation mark thought to control origin licensing has been shown to be associated with 50% of origins, this enrichment being significant for origins activated early or in mid-S (Figure 4-A and Table 6). The dynamic association of replication origins with open chromatin marks, such as H3K9ac, H3K4me3 and H2AZ, was strong (and significant) for origins replicated early in S phase, whereas origins activated in the second part of S phase were less associated with such marks (Figure 4-A and Table 6). We also found that these marks tended to be absent from late-activated origins, such as K562 cells (Table 6). Overall, 64% of origins carried none of these three open chromatin, indicating that most origins may not be directly driven by the presence of open chromatin marks, as previously proposed [1], [32].

**Table 6:** Association of replication origins with chromatin marks.

	H4K20me1		Ezh2		H3K27me3		H3K9me3		H3K9ac		H3K4me3		H2AZ	
K562	obs	Exp.	obs	Exp.	obs	Exp.	obs	Exp.	obs	Exp.	obs	Exp.	obs	Exp.
1-early	66 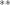	31	50 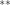	33	36 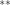	15	25 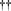	33	58 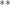	12	55 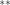	11	65 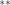	20
2-early	65 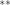	35	68 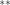	37	40 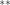	20	31 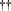	35	32 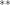	11	32 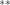	10	49 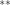	19
3-mid	58 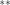	34	68 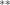	37	41 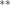	21	24 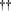	34	17 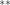	11	18 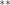	11	33 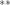	20
4-mid	45 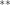	35	48 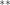	37	33 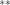	21	17 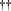	33	7 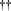	11	9 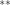	11	17 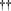	20
5-late	18 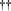	35	21 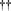	37	18 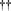	21	39 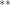	35	5 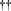	12	6 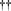	11	11 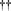	20
6-late	3 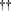	28	3 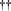	32	7 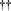	18	63 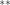	37	2 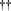	9	3 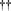	9	4 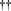	17

Chromatin mark coordinates were downloaded from the UCSC genome browser (ChIP-Seq peaks), and an origin of replication is associated with a given mark if the origin and the peak overlap. Expected (Exp.) percentage and significance of association (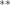 for significant enrichment, with 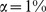 and 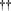 for significant depletion) were computed with random genomic segments sampled from mappable regions (see Materials and Methods).

The association with heterochromatin marks has been reported to be negatively correlated with replication timing. We, therefore, also investigated two histone marks known to be enriched in facultative and constitutive heterochromatin. Early origins displayed a significant depletion of H3K9me3, whereas late origins were characterized by a significant enrichment in this mark (Figure 4-A and Table 6). These results were confirmed by an independent study defining chromatin states (Table 5, HMM13). By contrast, we found that origins activated early and in mid-S phase were enriched in H3K27me3, which was thus associated with a large proportion of replication origins (40%) (Figure 4-A and Table 6). The association of this mark, deposited by PRC2 complexes, is confirmed by the strong overlap between H3K27me3 and Ezh2 responsible for the deposition of this mark (Table 6). These results were also confirmed by an independent study in which the polycomb-repressed chromatin state was annotated (Table 5), although the overlap with replication origins was weaker in this case.

We further focused on spatial interactions between marks that might characterize the temporal progression of replication. For each origin detected in K562 cells, we considered its linear distance to the closest mark, H2AZ, H4K20me1, H3K27me3, H3K9me3, H3K9ac or H3K4me3. We then used a discriminant analysis to identify combinations of chromatin marks that could discriminate (and thus characterize) early, mid- and late S-phase origins on the basis of their spatial co-localizations with replication origins. A complete description of the discriminant analysis is provided in the Methods section. A first combination of marks was characterized by the proximity of early origins to open chromatin marks (H2AZ, H3K9ac and H3K4me3) and H4K20me1. The distance between early origins and open marks increased with the progression of replication, whereas mid-S phase origins remain strongly associated with H4K20me1. Mid-S phase origins were also characterized by a strong association with H3K27me3, and the coupling of H4K20me1 and H3K27me3 with the exclusion of other marks constituted a strong characteristics of this category of origins. Finally the association with H3K9me3 was identified as characteristics of late origins, further from H4K20me1 and H3K27me3.

### H4K20me1 and H3K27me3 as potential regulators of replication

**Figure 5:**
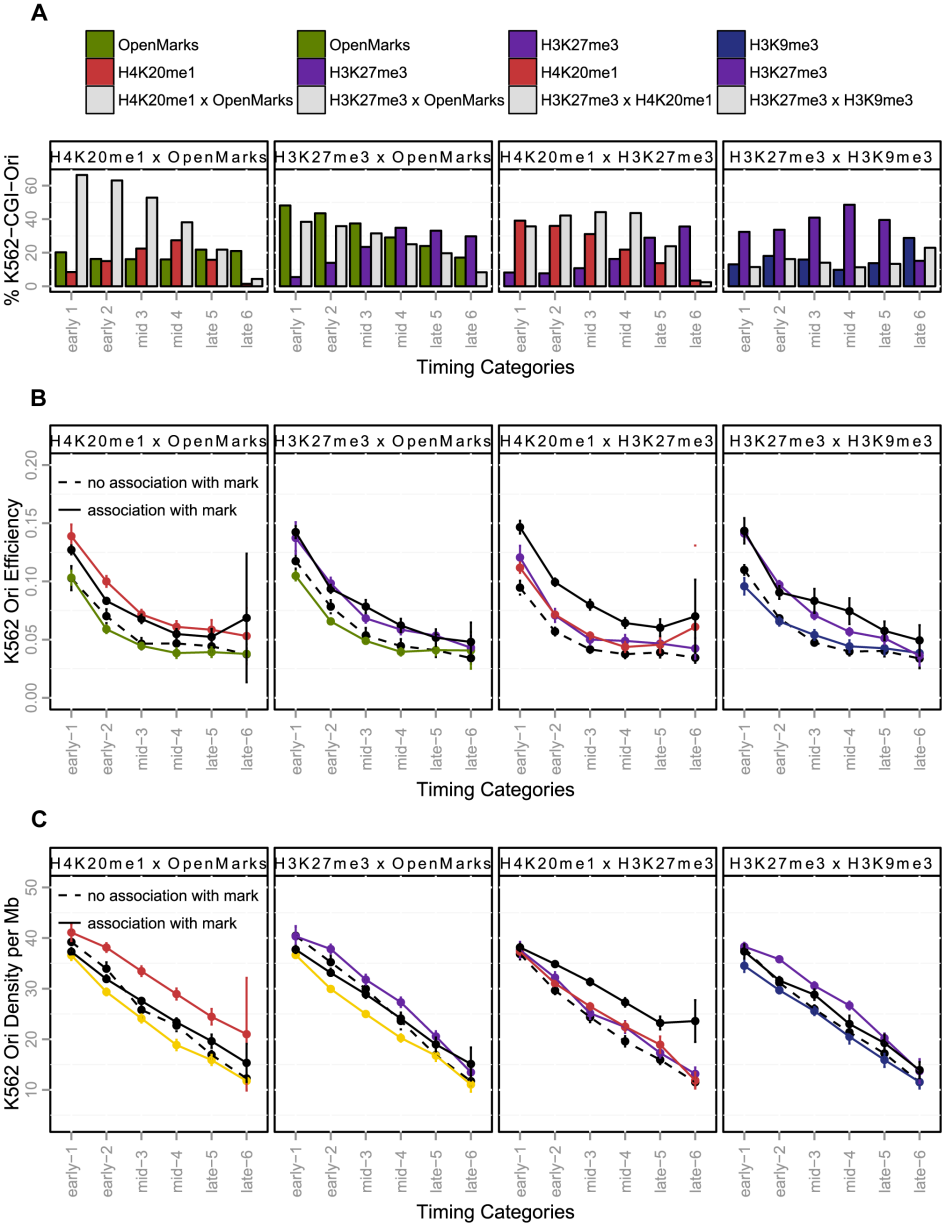
Effect of the coupling of histone marks and CGI-origins on origin efficiency and density (K562). A: Percentage of CGI-origins associated with chromatin marks, according to timing categories (from early to late origins, as explained in the Methods Section). The colored bars indicate the percentage of CGI-origins associated with one mark only, and grey bars represent the proportion of CGI-origins carrying both marks simultaneously. B: Variations of origin efficiency (as defined by the number of reads divided by the length of the origin) according to replication timing. C: Variation of origin density (as defined by the number of origins per megabase) with the timing of replication. Colored lines indicate origins associated with a single mark; solid black lines represent origins carrying both marks simultaneously, and black dotted lines represent origins that do not carry the marks considered. The values shown are mean values with 95% confidence intervals. Interactions between marks are significant when the solid black line is above the colored line. OpenMarks indicates origins associated with H2AZ, H3K9ac or H3K4me3.

Once we had elucidated the spatiotemporal interactions between origins and histone modifications, we further investigated whether they were associated with functional effects such as efficiency, length and density (Figure 4). We first investigated the responses to separate associations, and then studied the effect of combinations of marks. The separate analysis identified H4K20me1 and H3K27me3 as potential regulators of the replication program. When associated with CGIs, origins carrying these marks were characterized by a higher efficiency and length (Figure 4-B, C and Supp. Table S4), suggesting that they were associated with a larger number of initiation events. Colocalization with H4K20me1 and H3K27me3 was also associated with a higher density of origins (Figure 4-D, Supp. Table S5). By contrast, when associated with open marks, origins were less dense (Figure 4-D, Supp. Table S4), but their efficiency and length were not affected (Figure 4-B–C, Supp. Table S4), the slight effect on origin length observed in early S-phase being due to a high proportion of origins carrying both open chromatin marks and H4K20me1, as shown in Figure 5-A.

We then characterized the functional responses associated with marks combinations that we identified for early, mid-S phase and late origins. We found that H4K20me1 and open chromatin marks co-localize on 38% of early origins and 16% of for mid S-phase origins, this proportion being increased for CGI origins to 64% of early and 48% of mid-S phase origins, (Figure 5-A, Supp. Figure S4 for non-CGI origins). Moreover, H4K20me1 also colocalized with H3K27me3, particularly in origins activated in mid-S phase (Figure 5-A). These highly frequent colocalizations of marks were associated with different functional responses, as the coupling between H4K20me1 and H3K27me3 was the only combination to be associated with a significant increase in efficiency and density whatever the timing of replication (Figure 5-B–C, Supp. Table S5). The colocalization of H4K20me1 with open chromatin marks had very moderate additional effect over and above the separate effects of each mark (Figure 5-B–C, Supp. Table S5). The presence in ∼60% of origins of H4K20me1 or H3K27me3 (or both), and the strong functional responses associated with the colocalization of these marks suggests their potential importance in the control of the human genome replication program.

## Discussion

We aimed to identify sets of replication origins in a reliable manner, paying particular attention to the development of tools with good specificity and sensitivity. By comparing raw datasets obtained in two independent laboratories applying the same protocol to five different cell lines, we validated the SNS enrichment method, showing it to be highly reproducible. Moreover, the comparison of SNS-based detections with bubble trapping-based detections shows the validity of genome-wide detections of replication origins by two independent protocols. By applying this method to the data obtained for five different cell lines, we identified a subclass of constitutive origins common to all five cell lines, constituting a very robust set of human replication origins. These origins were enriched in CGIs and almost all of the CGI-Oris found in a given cell type were found to be constitutive.

### Identification of a consensus *cis*-element in replication origins

Origins overlapping with a CGI tended to be more efficient than non-CGI origins and were more abundant than would be expected on the basis of chance among the origins active in early S phase. This constitutes a subclass of origins playing an important role in establishing the spatiotemporal program of DNA replication. One key issue to be resolved concerns the way in which origins of this type are regulated. We investigated the characteristics making CGI active origins, by focusing on the differences between CGIs associated with origins (Ori-CGIs) and CGIs that were not associated with an origin (nonOri-CGIs). We found that Ori-CGIs were enriched in potential G4s L1–7, suggesting that G4s might be important cis-regulators of origin activity. This hypothesis was also supported by the observation that nonCGI-Oris also overlapped strongly with G4s. However, not all G4s are origins, suggesting that G4s are therefore not sufficient to induce the formation of an efficient origin.

We also performed genetic studies on one model origin, which confirmed that a structured G4 was important for origin activity and that this structured G4 had to cooperate with a 200 bp flanking *cis*-regulatory element to form a functional origin [26]. The cooperation with a flanking *cis*-module identified in one model origin added complexity to the origin signature, accounting for G4s not being systematically associated with origin function. We predict that the cooperating *cis*-module will act by binding transcription factors. Different classes of transcription factors may be involved, resulting in a complex signature motif for replication start sites. Taken together, our genome-wide and genetic studies and other published results [10], [18] suggest that G4s can be considered consensus cis-regulatory elements for replication origins in vertebrates. Further studies should search for trans-factors capable of recognizing structured G4s and, through this function, regulating origin function.

### Are there key histone marks involved in origin function?

We also deciphered the epigenetic characteristics of the temporal program of replication, providing new insight to improve our understanding of the spatiotemporal regulation of origins. Our work provides the first genome-wide demonstration of the strong association between early-firing origins and open chromatin marks. Our study was mainly based on origins detected in K562 cells, but we also provide similar analysis on HeLa cells (Supp. Figures S5, S6, S7, S8, Supp. Table S6). Early-replicated origins are enriched in open chromatin marks (they have more such marks than origins of other timing categories), consistent with the findings of previous genetic studies showing that the deposition of open chromatin marks close to replication origins can impose early firing in vertebrates [36], [37]. Moreover, a recent study showed that a strong replication origin lying within a region that is naturally replicated in late S phase may be induced to replicate earlier in S phase by the presence of binding sites for the USF transcription factor [37]. This shift is local, because replicons located 50 kb away are not affected, and it is associated with the appearance of open chromatin marks at the shifted origin. We also found that early origins are enriched in binding sites for transcription factors known to recruit open chromatin marks, including USF (data not shown). Overall, the results of genetic and genome-wide studies suggest that the deposition of open chromatin marks may be an important pathway for the regulation of early firing in vertebrates. We also found that early-replicated origins were the most efficient and that most were constitutive. We suggest that the construction of large early-replicated domains is dependent on the overlap of very efficient origins (CGI origins) and the recruitment of open chromatin marks by transcriptional regulatory factors, the binding sites of which are highly abundant in these domains.

We identified new associations with combinations of chromatin marks for origins replicated in mid-S phase regions, corresponding to the colocalization of the polycomb mark H3K27me3 with H4K20me1. A positive correlation between the PcG-mediated H3K27me3 mark and late replication has been reported in Drosophila [38], but no correlation has yet been established in mammalian cells, with the exception of one study that explored only 1% of the human genome, the findings of which conflicted with those of other genome-wide analyses [13]. However an association of H3K27me3 with mid- S phase-replicating chromosomal domains was recently identified, although a substantial correlation with early-replicating domains was also described [14]. Likewise, a recent study demonstrated a direct role for Pc-G proteins in the regulation of late replication in Drosophila [39], and studies have provided strong support suggesting that this mark is important for the control of DNA replication particularly in mid-S phase [40], [41]. One study showed that Pc-G-mediated chromatin assembly occurs during the post-mitotic G1 phase in human cells and that the depletion of Suz12 (the essential non catalytic subunit of the enzyme responsible for the trimethylation of H3K27) in G1 impairs the progression of cells in the following S phase and, particularly, in mid/late S phase [40]. In another study on mouse embryos, the depletion of components of the PRC1 complex (Ring1 and Rnf2), which recognizes H3K27me3, was shown to block DNA synthesis in most two-cell embryos. Based on the appearance of H2AX foci in two-cell embryos, the authors also concluded that most depleted embryos did not finish S phase, suggesting their arrest in S phase [41]. Our study is the first to demonstrate a strong genome-wide association of H3K27me3 with replication origins activated in mid-S phase, suggesting that this mark is important for the control of DNA replication in mid-S phase in vertebrates. Moreover, we found that origins associated with this mark were generally more efficient and were embedded in regions with a higher density of replication origins (Figure 4-B), suggesting a regulatory role of this mark in origin selection. Further studies should focus on the local effect of this mark on origin firing.

Our results also highlight a potential key role of the H4K20me1 chromatin mark. It has already been suggested that the histone H4 Lys 20 methyltransferase PR-Set7 regulates replication origins in mammalian cells, based on the observations that 1) the onset of licensing coincides with an increase in H4K20me1 at known replication origins, and 2) PR-Set7 is normally degraded in S phase and the PR-Set7 mutant insensitive to this degradation displays the maintenance of H4K20me1 at replication origins and repeated DNA replication [34]. It has recently been shown that the function of PR-Set7 is dependent on the further methylation of H4K20me1 by Suv4-20h [35]. Thus, the regulation and timing of H4K20me1/2/3 is critical for the accurate regulation of origin firing. H4K20me1 mark deposition is the primary and necessary event leading to the trimethylated state. We investigated the statistical association of this monomethylation with replication origins and found a very strong coincidence of this mark with origins, suggesting that many replication origins may have the potential to be controlled by this modification. We also showed that origins carrying this mark were associated with increased efficiency and were located in regions with a higher density of potential origins. The next step in our investigations of this regulation will be the mapping of H4K20me1/2 and 3, genome-wide, during the different phases of the cell cycle crucial for origin preparation, from early G1 to late S phase. This work will provide insight into the relationship between the dynamics of H4K20 methylation and origin function.

## Materials and Methods

### SNS preparation, sequencing, and data access

Short nascent strands were purified as previously described [32], but with minor changes to the protocol. We pooled fractions 18 to 24. These fractions contained single-stranded DNA molecules of various sizes, from 1.5 to 2.5 kb. We used 500 U of a custom-made 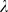-exonuclease (Fermentas (50 U.
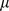
l-1)) for each preparation. For the genome-wide mapping of origins, eight SNS preparations were obtained independently from 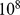 cells each and then pooled. SNS were made double-stranded by random priming with the Klenow exo-polymerase (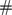 EP0421, Fermentas) and random primers (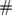
48190011, Invitrogen). Adjacent strands were then ligated with Taq DNA Ligase (M0208L, Biolabs). Two libraries were constructed with Illumina protocols and five deep sequencing runs were performed with a Solexa/Illumina GA I genome analyzer generating 75 bp reads. SOAP (v2) software was used to map reads to the reference human hg19 genome with the following parameters: r:0, I:30 and v:5 command-line. Data were deposited in the Gene Expression Omnibus (http://www.ncbi.nlm.nih.gov/geo/query/acc.cgi?token=pzexhssiceikczw&acc=GSE46189).

### Detection of replication origins with scan statistics

According to our detection model, read occurrences throughout the genome follow a Poisson distribution 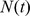 with a heterogeneous intensity 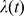 that can be interpreted as the coverage process. We also assume that at a given position t along the genome, the number of reads 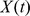 follows a geometric distribution 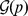. We then consider 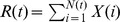, which counts the number of reads along the genome and, to detect local exceptional read accumulations, we compute 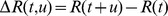, which quantifies the number of reads within a window of size u = 2 kb. For calculations of the the significance threshold for detection, we used scanning statistic results for compound distributions, making it possible to calculate the probability of the richest window actually being a false positive [15]. The detection is performed at level 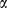 by setting threshold 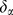 such that 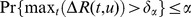. To account for coverage heterogeneities, we segment the coverage process (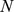) into regions of constant intensities (constant 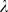). We use a segmentation model for this purpose, based on the Poisson distribution adapted from segmentation models for array CGH data analysis [42]. This segmentation step has two main advantages: First, it automatically detects regions of constant coverage (constant 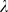) and regions with extremely low coverage that are excluded from the study. Second, it allows our significance thresholds to adapt to coverage variations. An example of detection is provided in Figure 1-A. Our method is available at http://pbil.univ-lyon1.fr/members/fpicard/research.html. Threshold 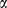 was calibrated using independent input DNA from public databases since input DNA was not available at the time of the experiment. We applied the detection method to input DNA and we assessed the percentage of nucleotides detected as origins in the SNS data that were also detected as peaks in the input DNA data (Supp. Table S2). We chose 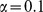 which corresponds to an estimated false discovery rate of 4%, 10% and 18% for K562, IMR90, and HeLa cells. This constitutes an overestimation of the false discovery rate of detection since origins detected in peak-assigned regions of the input-DNA are not necessarily false positives.

### Genomic and chromatin feature extraction

TSS, CpG islands, and chromatin mark positions were downloaded from the UCSC Genome Browser (http://genome.ucsc.edu/). Details on the datasets are provided in Supp. Table S3. The positions of G-quadruplexes were determined by applying Quadparser on hg19 [43], specifying the length of the spacer between 4 tracks of GGG or CCC with spacer of size 1–7, 1–15, 1–20, and 1–30. All results and tables can be downloaded from http://pbil.univ-lyon1.fr/members/fpicard/research.html.

### Determining mean replication timing profiles

We determined the mean replication timing profiles throughout the complete human genome from Repli-Seq data [19], [21], as previously described [44]. Repli-Seq tags for six FACS fractions were downloaded from the NCBI SRA website (study accession number: SPR0013933) for the erythroid K562 cell line, and from the UCSC ENCODE website http://hgdownload.cse.ucsc.edu/goldenPath/hg19/encodeDCC/wgEncodeUwRepliSeq/for the IMR90 fetal lung fibroblast cell line. For the HeLa cell line, we calculated the mean replication timing (MRT) rather than the S50 (median replication timing) [19], [20]. Timing categories were determined by dividing timing values into 6 intervals (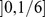, 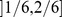, early origins, 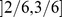, 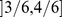, mid-S origins, 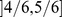, 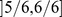, late origins).

### Randomization procedure

We used a randomization procedure to assess the expected overlap between origins of replications detected by SoleSearch, scan, and by bubble trapping. To compute the expected overlap between SNS-SoleS and SNS-scan origins (SoleS in scan, Table 2), we randomly sampled genomic intervals on the mappable fraction of the human genome that were excluding SNS-SoleS origins. The number of sampled intervals was the same as the number of SNS-SoleS origins, and we sampled 50 sets of such random origins. The overlap of SNS-scan with sampled intervals was used to assess the expected SoleS in scan overlap. To assess the scan in SoleS overlap, genomic intervals excluding SNS-scan origins were sampled. The procedure was similar to compute the expected overlap between Bubble and SNS origins.

We also used a randomization procedure to determine whether the association of replication origins with genomic features (such as CGIs, chromatin marks, Gquadruplex motifs) was significantly more frequent than would be expected by chance alone. For a given cell line, we compared the observed proportion of replication origins overlapping a given genomic feature with the expected proportion calculated from genomic intervals randomly sampled from throughout the genome. We excluded the replication origins we detected and the non mappable regions of the human genome as provided by the 1,000 Genomes Project [45], and we sampled 100,000 random intervals of the same length as origins of replication, on average. This procedure was repeated 1,000 times, to account for different sequence characteristics (such as gene density, or GC content). When replication timing was considered, the timing of replication for randomly sampled origins was determined from published timing data [21]. Random intervals were considered to be associated with genomic features if their intervals overlapped.

### Discriminant analysis for determining combination of marks

**Figure 6:**
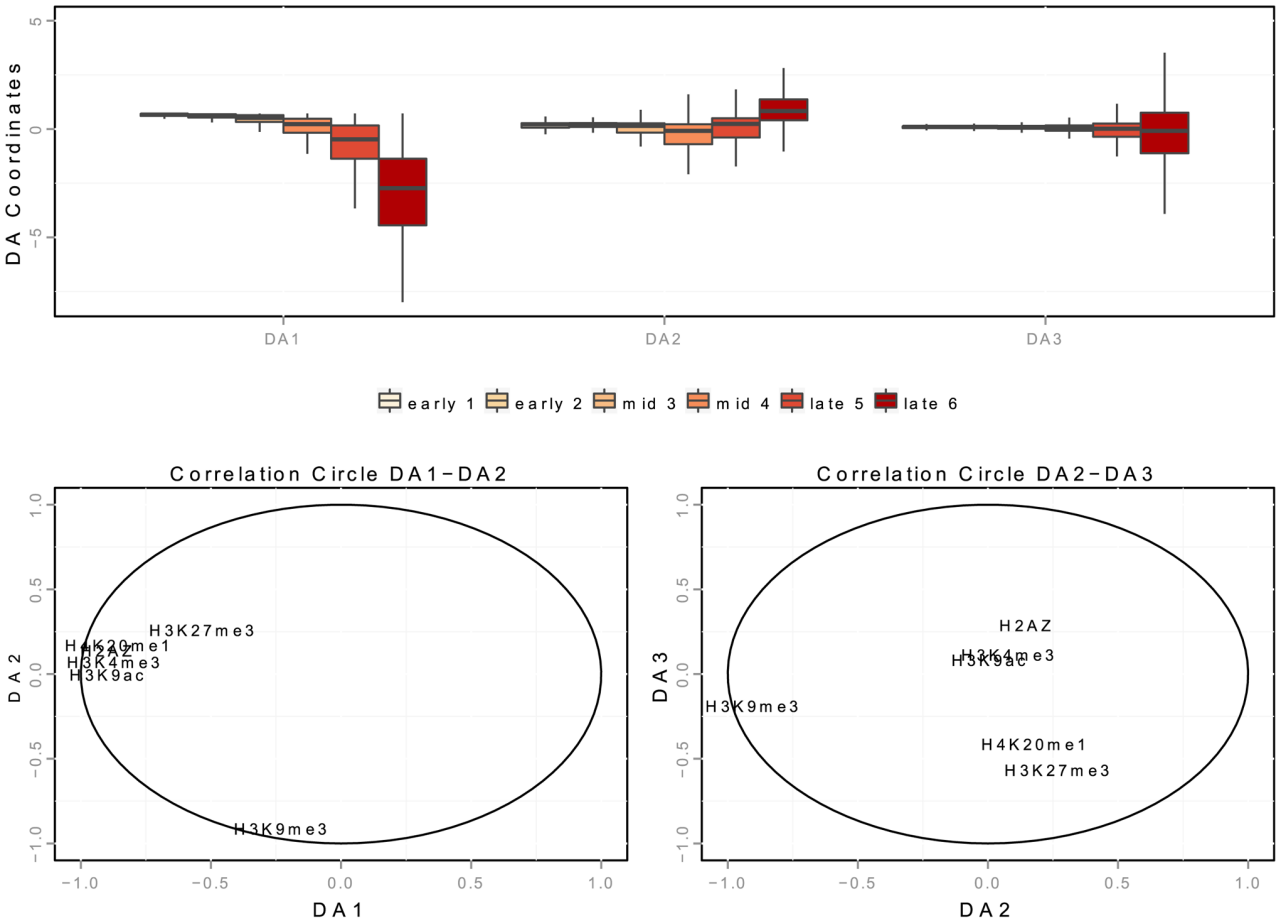
Spatial interaction of K562 replication origins with chromatin marks in discriminant analysis. A: boxplot of the 3 discriminant axis (DA) coordinates of origins according to timing categories (from early to late origins, as explained in the Methods Section). B,C: correlation circles of the three discriminant axis, with distances to chromatin marks. Correlations between discriminant axes and distances to chromatin marks are given in Table 7. High correlation means that the variables highly contribute to the creation of the axis.

We consider a linear discriminant analysis to find a linear combination of chromatin features which characterize early, mid-S phase and late origins [46]. We consider the data matrix with rows corresponding to origins detected in K562 cells and columns corresponding to linear distances to chromatin marks (datasets links are provided in Supp. Table S3). The interpretation of a discriminant analysis is based on two key ingredients (similarly to Principal Component Analysis): the position of the origins on the discriminant axis (Figure 6-A) and the correlations of the chromatin features with the discriminant axis (Figure 6-B,C). DA1 is the discriminant axis best discriminating between timing categories. It comprises the distances of origins to open chromatin marks (negative correlation with distances to H2AZ, H3k9ac, H3k4me3) and to H4k20me1/H3K27me3 (Table 7). This indicates that the temporal decrease observed along DA-1 (Figure 6-A) corresponded to an increase in the distance to these marks. Consequently, the first combination of chromatin marks that emerged was the proximity of open chromatin marks and H4K20me1 to early origins. The second axis (DA2) illustrates the opposition between H3K9me3 and other marks, and shows a different pattern between mid and late origins. Mid origins have a lower coordinate on DA2 (Figure 6-A), which corresponds to a smaller distance to H3K27me3/H4K20me1. DA2 was controlled by a positive correlation with the distance to H3K27me3/H4K20me1 (and to a lesser extent to H2AZ), along with a strong negative correlation with the distance to H3K9me3 (Table 7). Thus the proximity of origins to H3K27me3 and H4K20me1 emerged as a marks combination for mid-S phase origins. Finally late origins have a higher coordinate on DA2 (Figure 6-A), which corresponds to a smaller distance to H3K9me3.

**Table 7:** Correlation between discriminant axis and distance to chromatin marks on K562 cells.

	DA1	DA2	DA3
dist-H2AZ	−0.45	+0.19	+0.76
dist-H4K20me1	−0.47	+0.25	−0.50
dist-H3K27me3	−0.28	+0.24	−0.35
dist-H3K9me3	−0.12	−0.88	−0.05
dist-H3K9ac	−0.50	−0.26	−0.15
dist-H3K4me3	−0.49	−0.07	+0.15

Correlation coefficients between the distance of origins to chromatin marks and the discriminant axis (DA), as provided by the MixOmics Package [46]. Note that origins (detected on K562 cells) associated with chromatin marks correspond to distance 0 (strict overlap). Large distances therefore correspond to origins that are strictly not associated with a given mark. The sign of the correlation coefficient is important. If a discriminant axis is positively correlated with a distance, when the value along this axis increases the distance to the corresponding mark increases as well. Similarly, if it is negatively correlated, when the value along the axis decreases, the distance to the corresponding mark increases.

## Supporting Information

Figure S1 Snapshot of replication origins by different methods. Snapshot of the UCSC Genome Browser that visualizes origins of replication detected by bubble-trapping on GM06990 cells (top line), SNS-scan origins in HeLa cells (non-clustered and clustered). Last two lines correspond to SNS-SoleS origins [9] in HeLa cells (non-clustered and clustered).
(EPS)

Figure S2 Distribution of origins size for different cell lines. The distribution of origins size depend on the type of the origin (constitutive, common, specific).
(EPS)

Figure S3 Constitutive, common and cell specific replication origins (HeLa, IMR90) and association with CGIs. A: Percentage of constitutive/common/Hela-specific (ori IMR90-specific) origins in each timing category (from early to late origins, as explained in the Methods Section). Constitutive origins are determined by the intersection of the origins from the five cell lines -H9, HeLa, IMR90, K562 and iPS- with Galaxy [47]. B: Number of constitutive/common/HeLa-specific (or IMR90 specific) origins in each timing category, with distinction between origins associated and not associated with CGIs. Origins classified as CGIs correspond to origins strictly overlapping a CGI. Positions of CGIs are taken from UCSC Genome Browser annotation. C: Boxplots of origin efficiency according to timing, constitutive/common/HeLa-specific (or IMR90-specific) nature and association with CGIs. Origin efficiency is defined as the number of reads within the origin interval divided by the length of the origin (i.e. the density of reads within a given origin).
(EPS)

Figure S4 Effect of the coupling of histone marks and nonCGI-origins on origin efficiency and density (K562 cell line). A: Percentage of nonCGI-origins (origins that are not associated with CGIs) associated with chromatin marks, according to timing category (from early to late origins, as explained in the Methods Section). The colored bars indicate the percentage of nonCGI-origins associated with one mark only, and grey bars represent the proportion of nonCGI-origins carrying both marks simultaneously. B: Variations of origin efficiency (as defined by the number of reads divided by the length of the origin) according to replication timing. C: Variation of origin density (as defined by the number of origins per megabase) with the timing of replication. Colored lines indicate origins associated with a single mark; solid black lines represent origins carrying both marks simultaneously, and black dotted lines represent origins that do not carry the marks considered. The values shown are mean values with 95% confidence intervals. Interactions between marks are significant when the solid black line is above the colored line. OpenMarks indicates origins associated with H2AZ, H3K9ac or H3K4me3.
(EPS)

Figure S5 Effect of histone marks associations on origins efficiency, length and density (HeLa). A: Percentage of origins associated with chromatin marks according to timing categories (from early to late origins, as explained in the Methods Section) and CGI association. B: Variations of origin efficiency (as defined by the number of reads divided by the length of the origin) with replication timing, mark associations, and association with CGIs. C: Variations of origin length in kb with replication timing, mark associations, and association with CGIs. D: Variations of origins density (as defined by the number of origins per Megabase) with replication timing, mark associations, and association with CGIs. Values correspond to mean values with 95% confidence intervals. A comparison of the solid and dashed lines highlights the effect of associations of marks. OpenMarks indicates origins associated with either H2AZ, H3K9ac or H3K4me3.
(EPS)

Figure S6 Effect of the coupling of histone marks and CGI-Origins on origin efficiency and density (HeLa). A: Percentage of CGI-origins associated with chromatin marks, according to timing categories (from early to late origins, as explained in the Methods Section). The colored bars indicate the percentage of CGI-origins associated with one mark only, and grey bars represent the proportion of CGI-origins carrying both marks simultaneously. B: Variations of origin efficiency (as defined by the number of reads divided by the length of the origin) according to replication timing. C: Variation of origin density (as defined by the number of origins per megabase) with the timing of replication. Colored lines indicate origins associated with a single mark; solid black lines represent origins carrying both marks simultaneously, and black dotted lines represent origins that do not carry the marks considered. The values shown are mean values with 95% confidence intervals. Interactions between marks are significant when the solid black line is above the colored line. OpenMarks indicates origins associated with H2AZ, H3K9ac or H3K4me3.
(EPS)

Figure S7 Effect of the coupling of histone marks and nonCGI-origins on origin efficiency and density (HeLa). A: Percentage of nonCGI-origins associated with chromatin marks, according to timing categories (from early to late origins, as explained in the Methods Section). The colored bars indicate the percentage of CGI-origins associated with one mark only, and grey bars represent the proportion of CGI-origins carrying both marks simultaneously. B: Variations of origin efficiency (as defined by the number of reads divided by the length of the origin) according to replication timing. C: Variation of origin density (as defined by the number of origins per megabase) with the timing of replication. Colored lines indicate origins associated with a single mark; solid black lines represent origins carrying both marks simultaneously, and black dotted lines represent origins that do not carry the marks considered. The values shown are mean values with 95% confidence intervals. Interactions between marks are significant when the solid black line is above the colored line. OpenMarks indicates origins associated with H2AZ, H3K9ac or H3K4me3.
(EPS)

Figure S8 Spatial interaction of HeLa replication origins with chromatin marks in discriminant analysis. A: Boxplot of the 3 discriminant axis (DA) coordinates of origins according to timing categories (from early to late origins, as explained in the Methods Section). B–C: correlation circles of the three discriminant axis, with distances to chromatin marks. Correlations between discriminant axes and distances to chromatin marks are given in Supp. Table S6. DA1 is the discriminant axis best discriminating between timing categories; it comprises the distances of origins to open chromatin marks (negative correlation with distances to H2AZ, H3k9ac, H3k4me3) and to H4k20me1 (Supp. Table S6). Consequently, the association with open chromatin marks is stronger for origins firing early in S phase than for those firing later. The second axis illustrates the influence of H4K20me1-H3K27me3-H3K9me3 and their opposition with other marks as show in correlation circle DA2–DA3 (in particular for the effect of H2AZ).
(EPS)

Table S1Technical reproducibility of scan-based origins. Percentage of overlaping peaks detected in 5 different technical replicates (K562 cells). Pair-wise comparisons show a 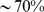 of concordance between replicates.(TXT)

Table S2 Calibration of the detection threshold for the scan method using external input-DNA. The scan detection method was applied to input-DNA only, and we computed the number of peaks that overlaped with peaks detected in the SNS data. Here, a “false positive” peak is a peak that is detected in the SNS data and also in the input data. A “true positive” peak is a peak that is detected in the SNS data and not in the input data. We propose an estimation of the false discovery rate that corresponds to the proportion of peaks detected in the SNS data that are also detected in the input data.
(TXT)

Table S3 Links to the genomic and chromatin data.
(TXT)

Table S4 Statistical testing of chromatin marks effects on origins efficiency, length and density (K562 cells). A t-test is performed for each timing category, to compare the efficiency of origins between origins associated with a mark (asso) and not associated with a mark (non asso). Efficiency is defined as the number of reads within the detected origins.
(TXT)

Table S5 Statistical testing of combinations of chromatin marks effects on origins efficiency, and density (K562 cells). A t-test is performed for each timing category, to compare the efficiency of origins between origins associated with a combination of marks (interaction) and associated with a mark (marginal effect). Efficiency is defined as the number of reads within the detected origins.
(TXT)

Table S6 Correlation between discriminant axis and distance to chromatin marks on HeLa cells. Correlation coefficients between the distance of origins to chromatin marks and the discriminant axis (DA), as provided by the MixOmics Package [47]. Note that origins (detected on HeLa cells) associated with chromatin marks correspond to distance 0 (strict overlap). Large distances therefore correspond to origins that are strictly not associated with a given mark. The sign of the correlation coefficient is important. If a discriminant axis is positively correlated with a distance, when the value along this axis increases the distance to the corresponding mark increases as well. Similarly, if it is negatively correlated, when the value along the axis decreases, the distance to the corresponding mark increases.
(TXT)
